# Fetal microglial phenotype *in vitro* carries memory of prior *in vivo* exposure to inflammation

**DOI:** 10.3389/fncel.2015.00294

**Published:** 2015-08-04

**Authors:** Mingju Cao, Marina Cortes, Craig S. Moore, Soo Yuen Leong, Lucien D. Durosier, Patrick Burns, Gilles Fecteau, Andre Desrochers, Roland N. Auer, Luis B. Barreiro, Jack P. Antel, Martin G. Frasch

**Affiliations:** ^1^Department of Obstetrics and Gynaecology, Faculty of Medicine, CHU Ste-Justine Research Centre, Université de MontréalMontréal, QC, Canada; ^2^Department of Neurosciences, Faculty of Medicine, CHU Ste-Justine Research Centre, Université de MontréalMontréal, QC, Canada; ^3^Faculty of Veterinary Medicine, Animal Reproduction Research Centre, Université de MontréalMontréal, QC, Canada; ^4^Neuroimmunology Unit, Montréal Neurological Institute, McGill UniversityMontréal, QC, Canada; ^5^Department of Clinical Sciences, Faculty of Veterinary Medicine, Université de MontréalQC, Canada; ^6^Département de Pathologie, University Hospital Ste-Justine, Université de MontréalQC, Canada; ^7^Department of Pediatrics, Faculty of Medicine, CHU Ste-Justine Research Centre, Université de MontréalMontréal, QC, Canada

**Keywords:** brain, neuroinflammation, bioinformatics, RNAseq, sheep, metabolism, cytokines, epigenetics

## Abstract

**Objective:** Neuroinflammation *in utero* may result in life-long neurological disabilities. The molecular mechanisms whereby microglia contribute to this response remain incompletely understood.

**Methods:** Lipopolysaccharide (LPS) or saline were administered intravenously to non-anesthetized chronically instrumented near-term fetal sheep to model fetal inflammation *in vivo*. Microglia were then isolated from *in vivo* LPS and saline (naïve) exposed animals. To mimic the second hit of neuroinflammation, these microglia were then re-exposed to LPS *in vitro*. Cytokine responses were measured *in vivo* and subsequently *in vitro* in the primary microglia cultures derived from these animals. We sequenced the whole transcriptome of naïve and second hit microglia and profiled their genetic expression to define molecular pathways disrupted during neuroinflammation.

**Results:** *In vivo* LPS exposure resulted in IL-6 increase in fetal plasma 3 h post LPS exposure. Even though not histologically apparent, microglia acquired a pro-inflammatory phenotype *in vivo* that was sustained and amplified *in vitro* upon second hit LPS exposure as measured by IL-1β response *in vitro* and RNAseq analyses. While NFKB and Jak-Stat inflammatory pathways were up regulated in naïve microglia, heme oxygenase 1 (*HMOX1*) and Fructose-1,6-bisphosphatase (*FBP*) genes were uniquely differentially expressed in the second hit microglia. Compared to the microglia exposed to LPS *in vitro* only, the transcriptome of the *in vivo* LPS pre-exposed microglia showed a diminished differential gene expression in inflammatory and metabolic pathways prior and upon re-exposure to LPS *in vitro*. Notably, this desensitization response was also observed in histone deacetylases (*HDAC*) *1, 2, 4*, and *6*. Microglial calreticulin/LRP genes implicated in microglia-neuronal communication relevant for the neuronal development were up regulated in second hit microglia.

**Discussion:** We identified a unique *HMOX1*_down_ and *FBP*^up^ phenotype of microglia exposed to the double-hit suggesting interplay of inflammatory and metabolic pathways. Our findings suggest that epigenetic mechanisms mediate this immunological and metabolic memory of the prior inflammatory insult relevant to neuronal development and provide new therapeutic targets for early postnatal intervention to prevent brain injury.

## Introduction

Brain injury acquired antenatally remains a major cause of long-term neurodevelopmental sequelae (Saigal and Doyle, 2008). There is growing clinical and experimental evidence for maternal and fetal infection acting via systemic and neuroinflammation to cause fetal brain injury or contributing to *in utero* asphyxial brain injury with consequences for postnatal health (Hagberg et al., 2002; Rees and Inder, 2005; Wang et al., 2006; Gotsch et al., 2007; Murthy and Kennea, 2007; Fahey, 2008).

In humans, the main cause of fetal inflammation is chorioamnionitis, a frequent condition affecting 10% of all pregnancies and up to 40% of preterm births. Chorioamnionitis is associated with ~nine-fold increased risk for cerebral palsy spectrum disorders with life lasting neurological deficits and an increased risk for acute or life-long morbidity and mortality (Fahey, 2008; Agrawal and Hirsch, 2012; Fishman and Gelber, 2012).

In addition to short-term brain damage, neuroimmune responses to *in utero* infection may also have long-term health consequences, the “second hit” hypothesis: In adults, exposure to inflammatory stimuli can activate microglia (glial priming, reviewed in Billiards et al., 2006; Karrow, 2006; Bilbo and Schwarz, 2009; Bilbo and Tsang, 2010; Ajmone-Cat et al., 2013; Bolton et al., 2014).

We hypothesized that an inflammatory response induced by lipopolysaccharide (LPS) will result in microglial activation reflecting neuroinflammation. To test the “second hit” hypothesis, we developed a protocol to culture fetal sheep microglia and re-expose them to LPS under *in vitro* conditions allowing a more mechanistic study of their phenotype.

## Materials and methods

### Ethics statement

This study was carried out in strict accordance with the recommendations in the Guide for the Care and Use of Laboratory Animals of the National Institutes of Health. The respective *in vivo* and *in vitro* protocols were approved by the Committee on the Ethics of Animal Experiments of the Université de Montréal (Permit Number: 10-Rech-1560).

### Anesthesia and surgical procedure

We instrumented pregnant time-dated ewes at 126 days of gestation (dGA, ~0.86 gestation) with arterial, venous and amniotic catheters and ECG electrodes (Frasch et al., 2007). Ovine singleton fetuses of mixed breed were surgically instrumented with sterile technique under general anesthesia (both ewe and fetus). In case of twin pregnancy the larger fetus was chosen based on palpating and estimating the intertemporal diameter. The total duration of the procedure was approximately 2 h. Antibiotics were administered to the mother intravenously (trimethoprim sulfadoxine 5 mg/kg body weight) as well as to the fetus intravenously and into the amniotic cavity (ampicillin 250 mg). Amniotic fluid lost during surgery was replaced with warm saline. The catheters exteriorized through the maternal flank were secured to the back of the ewe in a plastic pouch. For the duration of the experiment the ewe was returned to a metabolic cage, where she could stand, lie and eat *ad libitum* while we monitored the non-anesthetized fetus without sedating the mother. During postoperative recovery antibiotic administration was continued for 3 days. Arterial blood was sampled for evaluation of maternal and fetal condition and catheters were flushed with heparinized saline to maintain patency.

### *In vivo* experimental protocol

Postoperatively, all animals were allowed 3 days to recover before starting the experiments. On these 3 days, at 9.00 am 3 mL arterial plasma sample were taken for blood gasses and cytokine analysis. Each experiment commenced at 9.00 am with a 1 h baseline measurement followed by the respective intervention as outlined below. FHR and arterial blood pressure was monitored continuously (CED, Cambridge, UK, and NeuroLog, Digitimer, Hertfordshire, UK). Blood samples (3 mL) were taken for arterial blood gasess, lactate, glucose, and base excess (ABL800Flex, Radiometer) and cytokines at the time points 0 (baseline), +1 (i.e., after LPS administration), +3, +6, +24, +48, and +54 h (i.e., before sacrifice at day 3). For the cytokine analysis, plasma was spun at 4°C (4 min, 4000 g, Eppendorf 5804R, Mississauga, ON), decanted and stored at −80°C for subsequent ELISAs. After the +54 h (Day 3) sampling, the animals were sacrificed with an overdose of barbiturate (30 mg pentobarbital sodium, Fatal-Plus; Vortech Pharmaceuticals, Dearborn, MI) and a post mortem was carried out during which fetal gender and weight were determined. The fetal brain was then perfusion-fixed with 250 mL of cold saline followed by 250 mL of 4% paraformaldehyde and processed for histochemical analysis or dissected for cell culture (details see *in vitro* microglia culture paragraph). Fetal growth was assessed by body, brain, liver, and maternal weights.

Nine fetuses were used as controls receiving NaCl 0.9%. Twelve fetuses received LPS (400 ng/fetus/day) derived from *E. coli* (Sigma L5293, from *E. coli* O111:B4, ready-made solution containing 1 mg/ml of LPS) were administered intravenously to fetuses on days 1 and 2 at 10.00 am to mimic high levels of endotoxin in fetal circulation over several days as it may occur in chorioamnionitis.

### *In vitro* microglia culture protocol

**Figure 1:**
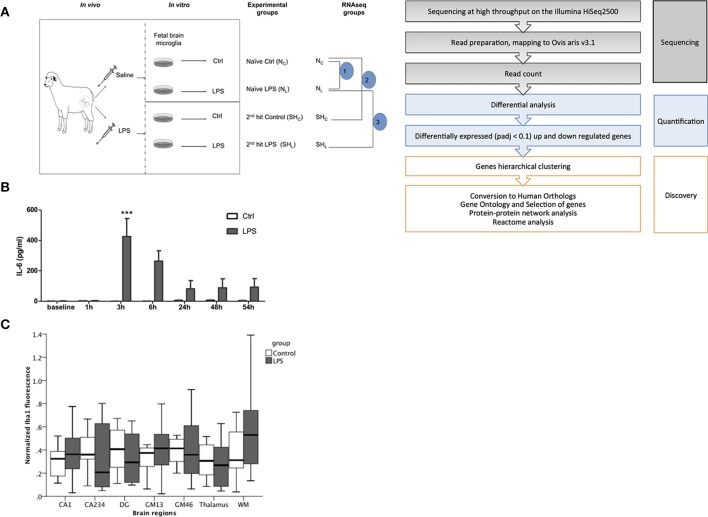
**Fetal sheep ***in vivo*** LPS exposure causes a systemic inflammation. (A)** Experimental design. *In vivo, in vitro* and RNAseq experiments are illustrated. *In vivo* study, Control (saline) and LPS; *in vitro* study, cultured cells derived from *in vivo* Control animal, named as Naïve, whereas cells derived from LPS-exposed animal named as 2nd hit (second hit, SH), there are four experimental groups: naïve Control (N_C_), naïve LPS (N_L_), 2nd hit control (SH_C_), and 2nd hit LPS (SH_L_), respectively. For RNAseq data comparisons, we first compared pair 1 (*n* = 6) Control (N_C_) vs. LPS-exposed naïve microglia (N_L_); then pair 2 (*n* = 4) naïve control (N_C_) vs. 2nd hit Control (SH_C_); and finally pair 3 (*n* = 4) naïve LPS-exposed microglia (N_L_) vs. 2nd hit LPS-exposed microglia (SH_L_). **(B)** IL-6 levels peaked at 3 h in fetal sheep plasma following LPS administration *in vivo* (^***^P < 0.001). *In vivo* Control group, *n* = 9, *in vivo* LPS group, *n* = 12. Blood samples were collected in heparinized syringe from fetal arterial catheter, plasma was obtained by centrifugation. A sheep specific IL-6 ELISA was performed to measure the cytokine levels. **(C)** No evidence of Iba1+ fetal *brain* microglia inflammatory response to LPS exposure *in vivo*. Normalized Iba1 + signal (microglia) fluorescence in six randomly chosen high power fields per brain region is shown in hippocampus [CA1, CA234, and dentate gyrus (DG) subregions], cortical gray matter (GM) layers GM13, GM46, white matter (WM), and thalamus. GEE model for prediction of Iba1+ normalized signal intensity: group main effect *p* = 0.62; brain region main effect *p* < 0.001; brain region^*^group interaction *p* = 0.13.

Fetal sheep brain tissues were obtained during sheep autopsy after completion of the experiment for *in vitro* study (Figure 1A). The non-instrumented, untreated twins were designated “naïve” (N_C_, no LPS exposure *in vivo*) and N_L_ when exposed to LPS *in vitro* for the first time. Instrumented animals that received LPS *in vivo* (SH_C_) were used for 2nd hit LPS exposure *in vitro* (SH_L_). Fetal sheep microglia culture protocol was adapted from an established human adult and fetal microglia culture protocol that was modified to include a myelin removal step following high-speed centrifugation (Durafourt et al., 2013). Briefly, fetal sheep cells were plated on poly-L-lysine (PLL)-coated tissue culture flasks at a concentration of 2 × 10^6^ cells /ml in DMEM with 5% heat-inactivated fetal bovine serum (Gibco, Canada Origin), 1% penicillin/ streptomycin, and 1% glutamine (5% DMEM), in which microglia are preferable to grow (Durafourt et al., 2013). Cells were allowed to incubate for seven days at 37°C, 5% CO_2_, followed by media change by centrifugation and addition of re-suspended cells back to the culture flask. Cells were continued to incubate for seven more days with 5% DMEM at 37°C, 5% CO_2_, before floating cells were collected. Carefully collecting the floating microglia to avoid contamination with astrocytes and oligodendrocytes, the cells were incubated in 24-well plates at 1 × 10^5^ cells/1.82 cm^2^ surface area with 1 mL of 5% DMEM for another 4–5 days, and then treated with or w/o LPS (100 ng/ml, Sigma L5024, from *E. coli* O127, B8) for 6 h. Cell conditioned media were collected for cytokine analysis, 0.5 ml TriZol were added per well for RNA extraction.

To verify microglia purity, a portion of floating cells were cultured in 24-well plates at above conditions for flow cytometry analysis, cell morphology was documented with light microscopy (see Supplementary Material). Another portion of floating cells were plated into Lab-Tek eight well chamber glass slide (Thermo Scientific) and treated with or w/o LPS for immunocytochemistry analysis, in this experiment, some wells of astrocytes cultured at DMEM with 10% FCS were included for comparison.

### Measurements of inflammatory responses

#### Measurement of cytokines in plasma and cell culture media

Cytokine concentrations in plasma (IL-6) and cell culture media (IL-1β) were determined by using an ovine-specific sandwich ELISA. Briefly, 96-well plates (Nunc Maxisorp, high capacity microtitre wells) were pre-coated with the capture antibody, the mouse anti sheep monoclonal antibodies (IL-6, MCA1659; IL-1β, MCA1658, Bio Rad AbD Serotec) at a concentration 4 μg/ml on ELISA plates at 4°C for overnight, after 3 times wash with washing buffer (0.05% Tween 20 in PBS, PBST), plates were then blocked for 1 h with 1% BSA in PBST for plasma samples or 10% FBS for cell culture media. Recombinant sheep proteins (IL-6, Protein Express Cat. no 968-305; IL-1 β, Cat. no 968-405) were used as ELISA standard. All standards and samples (50 μl per well) were run in duplicate. Rabbit anti sheep polyclonal antibodies (detection antibody IL-6, AHP424; IL-1β, AHP423, Bio Rad AbD Serotec) at a concentration of 4 μg/ml were applied in wells and incubated for 30 min at room temperature. Plates were washed with washing buffer for 5–7 times between each step. Detection was accomplished by assessing the conjugated enzyme activity (goat anti-rabbit IgG-HRP, dilution 1:5000, Jackson ImmunoResearch, Cat. No 111-035-144) via incubation with TMB substrate solution (BD OptEIA TMB substrate Reagent Set, BD Biosciences Cat. No 555214), color development reaction was stopped with 2 N sulphuric acid. Plates were read on ELISA plate reader at 450 nm, with 570 nm wavelength correction (EnVision 2104 Multilabel Reader, Perkin Elmer). The sensitivity of IL-6 ELISA for plasma was 16 pg/ml, the sensitivity of IL-1b ELISA for media was 41.3 pg/ml, respectively. For all assays, the intra-assay and inter-assay coefficients of variance was <5 and <10%, respectively.

#### Immunofluorescence imaging analysis

**Figure 2:**
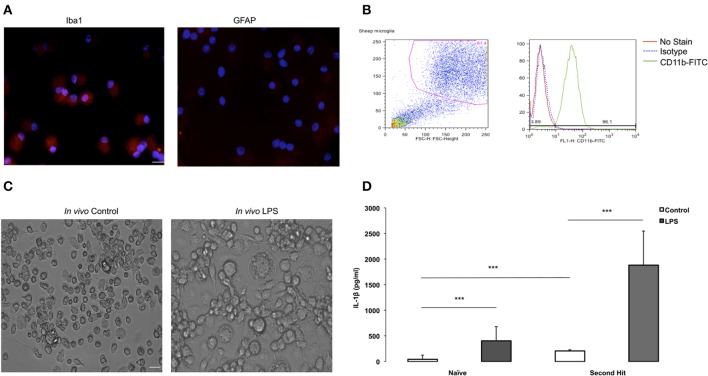
**Purity validation of fetal sheep brain primary microglia in cultures and LPS second hit**. **(A)** Photomicrographs (ICC) confirming cell purity. Iba1+ staining in microglia vs. undetectable GFAP signal in microglia indicating no contamination with astrocytes in the culture. Microglia were cultured in eight-well chamber slide with DMEM +5% FCS for 4–7 days. Microglia were collected from the floating fraction and stained for Iba1 and GFAP. Scale bar = 50 μm. Magnification 40× for both images. **(B)** Purity of fetal sheep brain primary microglia cultures was verified by flowcytometry. After several days in culture, fetal sheep microglia were scraped from the wells using a cell scraper and blocked for 30 min using normal mouse IgG and 10% human serum. Cells were then stained using a FITC-conjugated monoclonal bovine anti-CD11b (1:40, Bio-Rad) on ice for 20 min. Cells were washed in FACS buffer and analyzed using a BD FACSCalibur and FlowJo software. **(C)** Microglia from *in vivo* LPS exposed brain appear more aggregated *in vitro* than microglia derived from *in vivo* controls. Microglia were cultured in 24-well plates with DMEM +5% FCS for 4–7 days, when images were taken. Cells were extracted from a twin control fetal brain and an *in vivo* LPS exposed fetal brain (Magnification 20× for both images). **(D)** Effect of “second-hit” *in vitro* LPS treatment on microglial phenotype. BOTTOM: IL-1β concentration in conditioned media of microglia derived from fetal sheep brain that were exposed to LPS vs. saline *in vivo* (^***^P < 0.0001). Cultures from *in vivo* LPS-exposed (SH), *n* = 4, cultures from *in vivo* Control (Naïve), *n* = 10. Cell culture media supernatant was obtained by centrifugation upon cell culture termination. A sheep specific IL-1β ELISA was performed to measure cytokine levels in cell culture media. *In vitro*, at baseline, microglia secreted more IL-1β in the *in vivo* LPS group (SH_C_) than in naïve Control (N_C_). LPS re-exposure (SH_L_) further increased IL-1β vs. naïve LPS (N_L_) by ~4.6-fold.

Complete brain was taken from the fetus during necropsy after perfusion and immediately immersed in 4% PFA for 48–72 h. The tissue sample was then washed and stored in 1× PBS buffer changed daily for 3 days. Finally, the brain was stored in 70% ethanol until further processing. All the brain tissue samples were kept at 4°C when they were in liquid. The fetal brains were cut into two equal halves of left and right hemispheres, and then sliced coronally and placed into cassettes to be processed with Leica TP 1020 Automatic Tissue Processor (Leica Instruments, Mussloch, Germany). The tissues were embedded in paraffin with Leica EG 1160 Paraffin Embedding Center (Leica Instruments, Mussloch, Germany). Five-micrometer slices were obtained from slicing the embedded tissue samples with the Leica RM2145 Rotary Microtome (Leica Instruments, Mussloch, Germany), and mounted on the Fisherbrand Colorfrost Plus microscope slides (Fischer Scientific). The sectioned brain tissue samples went through de-paraffinization with CitroSolv (Fischer Scientific), 100, 95, 70, and 50% ethanol at room temperature, and antigen retrieval with 10 mM citrate buffer at pH 6 before being washed with water and 1× PBS, and blocked by Background Sniper Blocking Reagent (Biocare Medical, Cat. No BS966JJ). Then the sections were incubated with the primary antibody (Iba1, rabbit polyclonal antibody 4, 1:250 dilution, Wako, Cat No. 019-19741) for 1 h, followed by washing with 1× PBS and incubation with secondary antibody (Alexa Fluor 568 goat anti-rabbit IgG, 1:400 dilution, Life Technologies, Cat no A-11011) for 30 min in the dark. After that, the sections were washed again with 1× PBS, and the nuclei were counterstained with DAPI (1:4000 dilution, Sigma D-9564). Finally, the sections were cover-slipped with Fisherfinest Premium Cover glass (22 × 50-1, Fisher Scientific) and Fluoromount-G (SouthernBiotech, Cat no 0100-01) mounting medium, and viewed after 24 h of drying. Widefield fluorescence microscopy was performed on the stained brain tissue samples with a Zeiss Axiovert 200 M inverted microscope (Jena, Germany), at the magnification of 40× using a HBO100 mercury-arc lamp as a light source. The images were captured using a Zeiss Axiocam HRm (high-resolution monochrome) CCD (charged-coupled device) camera. Six high power field (HPF) images at 40× magnification were obtained for each animal. Multichannel imaging was used with the Iba1 channel and the DAPI channel for obtaining the pictures used for macrophage quantification. Appropriate ranges of color were selected showing positive contiguous cytoplasmic staining as a criterion for microglia cell count scoring which were then applied uniformly to calibrated images for all brain regions (Figure 2). Scoring was performed in a blinded fashion to experimental groups. To normalize for cell density Iba1+ signal over the whole area measured (100 sq micron) was divided by the respective optical intensity values for each HPF according to Lin et al. (2000).

### RNAseq approach

To extract and quantify RNA, total RNA was extracted from cultured microglia using TRIzol Reagent (Life Technologies). To obtain enough RNA, same treatment cells were pooled in one replicate. RNA quantity and quality (RNA integrity number, RIN) was determined by using a RNA Nano Chips (Agilent RNA 6000 Nano Chips) with Agilent 2100 BioAnalyzer. All samples had a RIN-value ranging from 6 to 8.5, except for one sample having RIN = 5.5 but an acceptable 84% of transcripts mapped, which did not affect the read count for this sample.

A total of eight samples from four set of replicates were selected for RNA sequencing at high throughput, of which three replicates were derived from *in vivo* control fetal sheep and one replicate was from *in vivo* LPS-exposed (second hit) fetal sheep. RNAseq libraries were prepared by using Illumina TruSeq RNA Sample Preparation v2 kit (Illumina) and quality control was performed on the BioAnalyzer. Single-end 50-bp sequencing was performed at high throughput on an Illumina HiSeq2500 at the CHU Ste-Justine Core Facility Sequencing Platform. Raw data and RNAseq data discussed in this publication were deposited on NCBI and are accessible online with the GEO accession number GSE71037.

#### RNAseq data analysis

##### Reads alignment to the reference genome

To maximize the amount of genes covered, raw data were mapped to the reference genome of the sheep *Ovis aris* v3.1 from NCBI and Ensembl (GCA_000298735.1) as transcriptome reference. Index of the reference fasta file were built with Bowtie2 (Langmead and Salzberg, 2012), we then trimmed the adaptor of the fastQ files with TrimGalore, and mapped reads to the reference with Tophat2 (Kim et al., 2013). From the aligned reads from Tophat2, the number of reads per gene were counted with HTseq and assembled into a matrix containing the read count of each gene per sample (Anders et al., 2015).

##### Normalization and transcriptome analysis

Among packages available to test for differential expression, DESeq2 provides methods suited for the use of replicates; it uses negative binomial generalized linear models and estimates dispersion and logarithmic fold changes. We used DESeq2 to normalize the dataset, generated log_2_-fold changes and adjacent *P*-values (*p*_adj_) and therefore, to find differentially expressed (DE) genes in microglia (Love et al., 2014). We first compared N_C_ to N_L_ to understand gene expression in naïve microglial cells after *in vitro* exposure to LPS. Then, due to the lack of replicates, we were not able to compare second hit microglial cells to their respective second hit control. Instead, we compared the genetic expression difference in response to a pre-exposure *in vitro* to LPS in N_C_ vs. SH_C_. Finally, we assessed genetic expression in SH_L_ compared to N_L_. A gene was considered differentially expressed (DE) if its adjacent *p*-value was strictly lower than 0.1. Pools of DE up and down regulated genes were clustered and visualized in heat maps, generated in *R* using the log_2_ normalized counts and the heatmap 0.2 method of the gplots library (Warnes et al., 2009).

##### Gene selection and Gene Ontology (GO)

The sheep genome is not yet supported by most gene ontology platforms, therefore, downstream analyses were performed with orthologs in the human genome Homo sapiens. ToppGenes and ToppCluster (Chen et al., 2009; Kaimal et al., 2010) were used to test for functional annotation enrichment analyses of biologic process and pathway with a false discovery rate correction of 0.05 (Franceschini et al., 2013). Gene Ontology was then performed with Gorilla and significant networks (*P* < 0.03) were selected for further discussion (Bauer et al., 2008; Eden et al., 2009).

### Quantitative real-time PCR analysis

The expression profiles of candidate genes were validated by real-time qRT-PCR. Total RNAs were subjected to cDNA synthesis using a QuantiTech Rev. Transcription Kit (Qiagen). *HMOX1* and *FBP* mRNA were quantified by qRT-PCR using a QuantiFast SYBR Green PCR Kit (Qiagen) with STRATAGENE 3000 P, mRNA relative expression was calculated by the 2^−ΔΔ*Ct*^ method over housekeeping gene *GAPDH* compared to baseline (Livak and Schmittgen, 2001). Sheep specific *HMOX1* primers were designed with primer3 (Untergasser et al., 2012) and *FBP* primers were designed using Integrated DNA Technologies online tool and listed in Table 1.

**Table 1:** **Primers of quantitative real time PCR analysis of HMOX1 and FBP**.

**Gene name**	**Forward**	**Reverse**
HMOX1	CACCAAGTTCAAGCAGCTGT	CAACCCTGCGAGAAATGTCC
FBP	CGAATGTGACGGGAGATCAA	GGCATGTTTGTCTTCTTCTGAC
GADPH	TGAGATCAAGAAGGTGGTGAAG	GCATCGAAGGTAGAAGAGTGAG

### Statistical analyses

Generalized estimating equations (GEE) modeling was used to assess the effects of LPS while accounting for repeated measurements on fetal blood gasses and acid-base status, plasma and *in vitro* cytokines, cardiovascular responses [AR(1) correlation matrix to account for temporal structure] and *in vivo* Iba1+ fluorescence (independent correlation matrix to deal with the spatial distribution of Iba1+ fluorescence across the brain regions). We used a linear scale response model with LPS and time or brain regions as predicting factors to assess their interactions using maximum likelihood estimate and Type III analysis with Wald Chi-square statistics. Correlation analysis was performed using Spearman correlation coefficient. SPSS Version 21 was used for these analyses (IBM SPSS Statistics, IBM Corporation, Armonk, NY). Significance was assumed for *p* < 0.05. Results are provided as means ± SEM. Not all measurements were obtained for each animal studied.

## Results

### *In vivo* studies

#### Cohorts' characteristics

Maternal venous blood gasses, pH, and lactate did not significantly change during the experiments and were within physiological range throughout the experiment for both groups. Maternal and fetal cohort's characteristics are summarized in Table 2. Gestational age at time of the experimental day 1 averaged 130 days ± 1.3 dGA (term 145 dGA). Overall, mother and fetus were considered healthy based upon a physical examination and laboratory data collected.

**Table 2:** **Maternal and fetal ***in vivo*** clinical characteristics**.

**Characteristics**	Maternal^*^	Fetal^**^
Averaged body weight (kg)	76±11	3.8±0.9
Gender: control group, male		5∕9
Gender: LPS group, male		4∕12
Parity: control group		7∕9
Parity: LPS group		3∕12
pO_2_ (mmHg)	54±6	20±1
pCO_2_ (mmHg)	41±2	52±2
pH	7.44±0.01	7.37±0.04
Lactate (mmol/L)	0.7±0.2	1.5±0.9
BE (mmol/L)	1.1±0.2	3.3±2.3

* *Values averaged over the course of the experiment, mean ± SEM*.

** *Baseline characteristics averaged for control and LPS groups*.

#### Clinical-chemical data

Clinical-chemical data, reported elsewhere, (Durosier et al., 2015) are summarized in Table 2 and were within physiological range for both groups. We found significant time^*^LPS interactions for pH (*P* = 0.03), pO_2_, pCO_2_, lactate, and BE (all *P* < 0.001).

#### Cardiovascular analysis

As reported (Durosier et al., 2015), we found time-LPS interactions for mean arterial blood pressure and fetal heart rate responses (*P* = 0.015 and *P* < 0.001, respectively).

#### Plasma cytokines response to LPS

*In vivo* LPS exposure resulted in a peak of IL-6 at 3 h compared to baseline. We detected time-LPS interaction for fetal plasma IL-6 (*P* < 0.001, Figure 1B).

#### *In vivo* LPS effect on neuroinflammation

To assess the effect of *in vivo* LPS exposure on neuroinflammation *in situ* we measured microglial activation as Iba1+ immunofluorescence signal. We found a significant brain region main effect (*p* < 0.001), but no group main effect (*p* = 0.62) and no significant brain region^*^group interaction (*p* = 0.13) (Figure 1C). Thus, *in vivo* LPS exposure did not cause any measurable neuroinflammation as can be seen with higher doses of LPS using the same microglia marker (Keogh et al., 2012; Kuypers et al., 2013).

Overall, fetuses responded to the *in vivo* LPS exposure with signs of moderate sepsis as evident by the changes observed with arterial blood gas, pH, lactate, but with no signs of cardiovascular decompensation. Despite the systemic response to the LPS challenge, we observed no signs of neuroinflammation *in vivo*.

### *In vitro* studies

Having established a moderate LPS-induced *in vivo* fetal systemic inflammation paradigm without overt neuroinflammation *in situ*, we next aimed to test the functional properties of microglia exposed to LPS *in vivo* in an *in vitro* setting allowing characterization of microglial cytokine secretion and transcriptome profiles in response to LPS.

#### Primary fetal sheep microglia culture

*In vitro* studies were conducted in primary cultures derived from six controls (naïve) and from two *in vivo* LPS-exposed animals (SH). We were able to perform 1–2 *in vitro* replicates per each animal depending on cell numbers obtained.

We identified oligodendrocytes and neurons in the initial cell isolation in addition to microglia and astrocytes (data not shown). To enrich for microglia we subjected the cells to a second step as detailed in Methods. To verify cell culture purity, we performed immunofluorescence staining with a microglia marker confirming that the isolated primary microglia was very high (Figure 2A), whereas an astrocyte marker, GFAP, was absent from the cell population. To further verify cell purity, flow cytometry CD11b-FITC antibody was performed resulting in 96% of cultured cells are CD11b+ (Figure 2B), further indicating that a highly pure microglia population was obtained.

We used the purified highly enriched microglia cultures to pursue the second hit paradigm, i.e., how these cells behave *in vitro* in dependence on previous *in vivo* LPS exposure (Figure 1A). We found that microglia from *in vivo* LPS exposed fetal brain differ in morphology, showing more aggregation or clumping compared to naïve microglia (Figure 2C). This finding indicates that these microglia might have already been activated by LPS exposure *in vivo*.

Next, we investigated cytokine secretion properties of these cells in the absence or presence of LPS. For IL-1β, we found that *in vitro* LPS administration resulted in increased IL-1β in microglia compared to control cell cultures; this IL-1β response was potentiated by 4.6-fold in cells derived from animals with *in vivo* LPS exposure: 1884 ± 481 pg/ml vs. 406.14 ± 193 pg/ml (all *p* < 0.001, Figure 2D). Moreover, even in the absence of LPS *in vitro*, at baseline, microglia from the *in vivo* LPS group secreted more IL-1β (208.1 ± 16.63 pg/ml vs. 44.97 ± 59.21 pg/ml) with the fold increase being concordant with the level of gene expression increase (all *P* < 0.001, Table 3). Other pro-inflammatory cytokines of interest such as IL-6 and TNF-α were undetectable in cell-conditioned media (ELISA data not shown). Our findings suggest that a pro-inflammatory microglial phenotype acquired during *in vivo* exposure to LPS is sustained *in vitro* (second hit paradigm).

**Table 3:** **Gene expression summary in naïve (one time exposure to LPS ***in vitro***) and second hit (exposed once ***in vivo*** and second time ***in vitro***) microglia**.

**Relevance**	**Gene name (Common name)**	**Naïve microglia**	**Second hit control**	**Second hit**
Activated mitochondrial biogenesis	SLC2A4	6.386	**8.911**	0.821
	PPARGC1A	4.842	**5.835**	1.466
Adipocytokine signaling pathway	CAMKK1	−1.110	0.511	−0.205
	CAMKK2	0.660	1.206	0.453
Adiponectin	ADIPOQ	2.422	**4.588**	1.137
Adrenoceptor alpha 1A	ADRA1A	2.572	**4.687**	−0.471
AMPK signaling pathway	PRKAA1	0.169	0.218	−0.643
	PRKAA2	2.776	**4.784**	1.774
	PRKAB1	−0.189	−1.319	−2.603
	PRKAB2	0.861	0.179	0.066
	PRKAG1	−0.379	**−3.249**	−2.168
	PRKAG2	−0.405	−1.194	−0.513
	PRKAG3	−1.329	0.099	−0.050
B-cell development and survival	TNFSF13B (BAFF)	2.562	−0.955	−1.823
Calcium binding protein 39	CAB39	0.279	**−1.520**	−1.583
	CAB39L	−0.076	−1.092	−1.106
Esterase enzyme	ACHE	−0.036	−0.978	−0.698
	BCHE	−0.475	**−3.197**	−1.782
Fractalkine/CX3CR1 axis and biological signature of microglial cells		CX3CR1	2.017	3.079	0.880
CX3CL1	0.618	−0.443	0.076
	ITGAM (CD11b)	0.130	−0.113	−1.083
	IL1B	7.578	**1.766**	0.726
Fructose-1,6-Biphosphate	FBP	−0.792	1.465	**4.057**
Gluconeogenesis and glucolysis	ALDOA	−0.106	−0.485	0.355
	ALDOB	−1.659	−1.842	−2.033
	ALDOC	−0.266	−0.717	0.328
	PFKP	−0.461	−0.228	0.686
	GPI	−0.940	**−2.001**	−0.363
Growth, proliferation, fate determination, development, immunity		ELAVL1	−0.252	−0.986	−0.895
CCNA1	2.574	**6.259**	2.854
	CCNA2	0.143	0.098	0.686
	IRF9 (p48)	0.407	**−1.286**	−0.641
	PIM1	3.108	−0.815	−2.762
	EP300 (CBP)	0.784	0.638	−1.094
	CREBBP (CBP)	0.881	0.752	−0.835
	CISH (CIS)	7.170	2.217	−2.097
HDAC genes: potential epigenetic regulators	HDAC1	**2.271**	0.145	0.676
	HDAC10	−0.242	−0.840	0.116
	HDAC11	−0.214	0.867	0.812
	HDAC2	−0.299	**−2.746**	−2.423
	HDAC3	0.045	−0.692	0.321
	HDAC4	1.292	1.502	−0.691
	HDAC5	−0.869	0.333	0.501
	HDAC6	**−0.688**	−0.126	−0.430
	HDAC7	−0.109	0.643	−0.486
	HDAC8	0.336	−0.889	−2.510
	HDAC9	0.732	1.556	−1.816
Increased FFA oxidation	CPT1A	−0.260	0.631	0.100
	CPT1B	0.496	1.140	0.304
	CPT1C	0.608	0.315	0.503
Inhibit cell growth and protein synthesis	RPS6KB1	−0.078	−1.659	−1.444
	RPS6KB2	−0.569	**−1.900**	−0.634
	EIF4EBP1	−0.822	**−2.609**	−1.533
	PPARG2	**−2.093**	**−2.107**	−0.656
Inhibit protein synthesis	EEF2	−0.005	0.210	0.421
	EEF2K	−0.394	0.208	−0.321
Initiators of the JAK-Stat pathway	JAK1	0.213	−1.404	−2.112
	JAK2	2.289	0.000	−1.430
	JAK3	**2.965**	**2.121**	−0.920
	TYK2	0.948	−0.296	−0.929
	STAT1	−0.136	−0.825	−0.563
	STAT2	1.276	−1.321	−2.852
	STAT3	0.660	**−2.050**	−0.889
	STAT5A	3.365	−0.188	−2.798
	STAT5B	1.554	−0.436	−1.093
Insulin signaling pathway	IGF1	1.601	2.264	1.177
	IGF1R	−0.125	−0.489	−0.838
	IRS1	1.241	**2.665**	0.344
	IRS4	4.420	**7.086**	1.953
Iron metabolism and/or anti-inflammatory	HMOX1	**−2.686**	**−4.462**	**−4.303**
	NRF-2	0.855	−1.235	−1.225
JNK/P38 MAPK	MAPK8 (JNK)	0.544	−2.918	−1.173
	MAPK9 (JNK)	−0.266	**−2.257**	−2.853
	MAPK10 (JNK)	1.492	3.024	−0.262
	MAPK12 (P38)	−0.905	−0.708	0.458
	MAPK13 (P38)	3.294	3.173	0.171
	MAPK14 (P38)	−0.848	−0.612	0.113
Leptin	LEP	5.033	**7.429**	−0.143
LRP phagocytosis signaling	LRP1B	4.522	**6.380**	0.336
	LRP2	4.860	**6.571**	1.410
	LRP6	**1.052**	**1.850**	0.157
Lymphocyte adhesion, T-cell costimulation	ICAM-1	4.055	−0.181	−2.801
Lymphoid-tissue homing	CCL21	18.917	20.916	0.368
	CCL19	5.439	5.328	−0.321
mTOR signaling pathway	RHEB	−0.257	**−3.516**	−2.292
	AKT1S1	0.908	−0.657	−1.496
	MTOR	−0.107	−0.234	−1.066
	RPTOR	0.298	0.361	−0.625
Myeloiesis and B-cell lymphopoiesis	CXCL12 (SDF-1alpha)	−0.545	1.552	0.268
NF-kB signaling and inflammation	RELB	1.503	−1.389	−1.227
	NFKB	**2.676**	−0.323	−0.200
	NFKBIA	**2.578**	**−1.934**	−1.546
	NFKB1 (p50)	2.569	−0.801	−2.673
	RELA (p65)	−0.031	**−2.098**	−2.268
	PTGS2	5.166	−0.609	−2.168
	TNF	4.990	0.028	−2.743
	PTGS2	5.166	−0.609	−2.168
	IL8	4.779	**−2.847**	−3.988
	IL1B	7.578	**1.766**	0.726
	TNFAIP3	2.628	−0.542	−3.162
Nitric oxide (NO) and NO production	NOS1	6.201	**8.416**	1.233
	NOS1AP	0.386	−0.599	−1.393
	NOS2	5.951	**3.039**	−1.958
	NOS3	**5.266**	**7.637**	1.112
P53	TP53	−0.554	−0.379	−1.539
	MDM4	3.489	−13.081	−1.120
PIK3-Akt signaling pathway	PIK3CA	0.018	**−1.584**	−1.561
	PIK3CB	1.793	−0.972	−1.750
	PIK3CG	1.950	1.106	−0.094
	PIK3R1	1.330	**−1.533**	−1.884
	PIK3R3	1.083	2.066	−1.507
	PIK3R5	4.343	**2.695**	−0.635
	PDPK1	0.915	0.946	−0.606
	AKT2	−0.335	−0.857	−1.328
	AKT3	0.350	0.104	−1.292
	TSC1	1.099	0.940	−0.386
	TSC2	0.219	0.587	1.182
Quality control	TGFBR1	0.327	0.158	−0.943
	TGFβ	−0.419	**−1.798**	−0.690
	GFAP	−1.044	−5.512	−3.709
	ITGAM (CD11b)	0.130	−0.113	−1.083
	CD40	4.656	**2.831**	1.352
	IBA1 (AIF1)	0.060	0.208	0.956
Serine/threonine kinase 11	STK11	**−0.178**	**−1.553**	−1.491
STE20-related kinase adaptor alpha	STRADA	**−0.536**	**−1.252**	−1.293
	STRADB	**−0.506**	**−2.816**	−3.582
Tak1 protein	MAP3K7	−0.057	−0.809	−2.136
Toll-like receptor 4	TLR4	0.807	**−2.451**	−3.749
	LY96 (MD-2)	1.213	0.263	0.340
	LBP	4.472	**5.990**	0.172
Transcription factors	C-JUN	0.795	**−1.539**	−2.385
	C-FOS (FOS)	**−3.072**	−1.635	−0.317
	NFKB	**2.676**	−0.323	−0.200
	CREB1	0.782	−0.228	0.190
	ATF4 (creb TF)	0.968	−0.319	0.163
	CEBPB (CEBP)	0.586	−0.993	−1.451

*Bold values correspond to a significant log_2_ fold change (p_adj_ < 0.1)*.

*Differential analysis of the count data was done with the DESeq2 package; up regulated genes are highlighted in red and down regulated genes are highlighted in blue. Values in the column “naïve cells” correspond to fold change from the naïve controls (N_C_) to LPS-exposed microglia (N_L_). In the same way, we compared second hit control (SH_C_) to the N_C_. Fold changes are summarized in the column “second hit control.” Values in the “second hit” column represent the fold change from N_L_ to second-hit, i.e., LPS re-exposed, (SH_L_) microglia*.

#### RNAseq approach

##### General overview of the whole transcriptome sequencing

To explore the genomic landscape of fetal sheep microglia, we sequenced the transcriptome of naïve and “second hit” microglia. As a quality control, we tested the expression levels of GFAP and TNFα across our three comparisons, and confirmed that all cells in our platform shared the same gene expression characteristics of microglia (Table 3). As a control measure for cell purity, our data confirmed the presence of TGF-β1 in each sample, as previously reported (Butovsky et al., 2014). To further confirm cell purity and the findings on protein level (ELISA), our transcriptome analysis showed a 1.654-fold increase of IL-1β (log_2_ = 0.726) between naïve and second hit LPS-exposed microglia (Table 3, respectively N_L_ and SH_L_).

**Figure 3:**
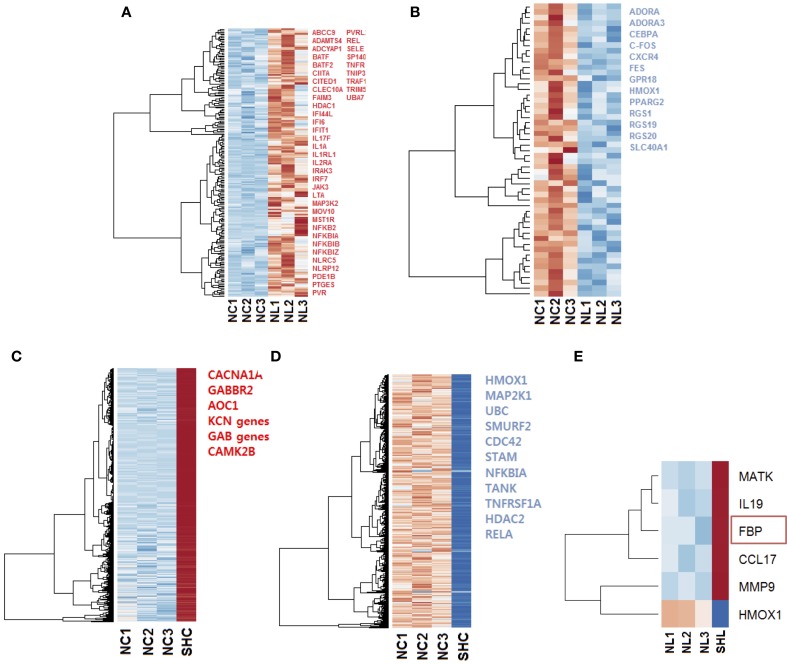
**Heat maps of the gene expression in microglia cells exposed to LPS**. Selected up regulated (red) and down regulated (blue) genes are listed; genes were selected with ToppCluster based on their significance in the immune response (log*P*>4.00) **(A)** Heat map of 205 differentially expressed (*p*_adj_ < 0.1) up regulated genes (red) in N_L_ microglia. **(B)** Heat map of 53 differentially expressed down regulated genes in N_L_ microglia, among selected genes indicated in blue, *HMOX1* was strongly differentially expressed (log_2_ = −2.686 and $p_{\text{adj}}=3.09\times 10^{-8}$). In both up and down regulated genes, we observed a different behavior for N_L3_ that did not affect our differential analysis. **(C)** Heat map of the 4112 most differentially expressed and up regulated genes in SH_C_ compared to N_C_ cells. Selected genes with ToppCluster (log*P*>4.00) include GABA receptor genes and genes related to the transport of ion, Calcium, Sodium, and Potassium. IL1B was up regulated with log_2_ = 1.766 ($p_{\text{adj}}=1.53\times 10^{-3}$) corresponding to a 3.40-fold increase **(D)** Among the 2530 most differentially expressed and down regulated genes identified, *HMOX1* was significantly down regulated (log_2_ = −4.462 and $p_{\text{adj}}=4.22\times 10^{-19}$). The reported genes were selected with ToppCluster (log*P* > 4.00) for their implication in the inflammatory response. **(E)** Differentially expressed up and down regulated genes in SH_L_ compared to N_L_ microglia. *HMOX1* was the only down regulated gene (log_2_ = −4.303, $p_{\text{adj}}=8.13\times 10^{-2}$). When comparing common genes in SH_C_ and SH_L_ cells, the gene *FBP* was unique to SH_L_ (red rectangle). NC, Naïve control microglia; NL, Naïve LPS-exposed microglia; SHC, Second hit control microglia; SHL, Second hit LPS-exposed microglia.

Firstly, we compared gene expression between the naïve controls and naïve LPS-exposed microglial cells. We found 258 differentially expressed genes (*p*_adj_ < 0.1), among which, 205 genes were up regulated and 53 were down regulated. We selected relevant differentially expressed genes with ToppCluster (log*P*>4.00) based on their role in the immune response (Figures 3A,B).

Then, to better understand the effect of an *in vivo* pre-exposure to LPS on biological processes, we compared gene expression between the naïve and second hit controls, i.e., N_C_ and SH_C_, respectively. We found 6642 differentially expressed genes, among which, we identified 4112 up regulated and 2530 down regulated genes. Selection of relevant genes with ToppCluster (log*P*>4.00) showed that up regulated genes in SH_C_ are mainly composed of *GABA* genes and genes responsible for calcium, potassium, and second messengers transport. Differentially expressed down regulated genes comprised the genes of the NF-κB signaling pathway and the *HMOX1* gene, responsible for iron metabolism (log_2_ = −4.462 and $p_{\text{adj}}=4.22\times 10^{-19}$).

**Figure 4:**
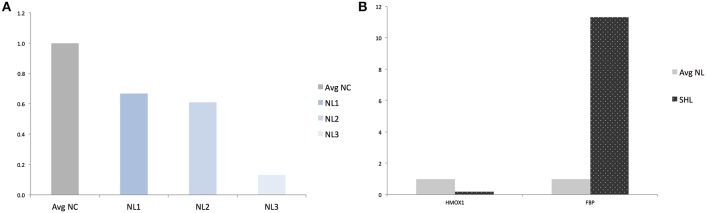
**Validation of RNAseq findings by qRT-PCR analysis of ***HMOX1*** and ***FBP***. (A)** Quantification of HMOX1 in each N_L_ microglia compared to averaged-N_C_. **(B)** Quantification of HMOX1 and FBP in SH_L_ compared to averaged-N_L_ microglia. Avg, Averaged; NC, Naïve control microglia; NL, Naïve LPS-exposed microglia; SHL, Second hit LPS-exposed microglia.

Finally, in an effort to discover the differences in response between N_L_ and SH_L_, we compared gene expression of the N_L_ set of three replicates and SH_L_ (*n* = 1). We identified a total of six differentially expressed genes: five were up regulated and one gene, *HMOX1*, was strongly down regulated (*HMOX1*_down_, log_2_ = −4.303 and $p_{\text{adj}}=8.13\times 10^{-2}$). Among the five differentially up regulated genes identified, Fructose-1,6-bisphosphatase (*FBP*) was uniquely differentially expressed in second hit LPS-exposed microglia (*FBP*^up^, log_2_ = 4.057 and $p_{\text{adj}}=9.40\times 10^{-2}$). The expression profile of *HMOX1* and *FBP* were assessed by quantitative real-time PCR (qRT-PCR). The results showed that the expressions of *HMOX1* and *FBP* were consistent with the expressions from the transcriptome analyses (Figures 4A,B). The roles of these genes are discussed below.

## Discussion

We established for the first time an *in vivo–in vitro* endotoxin double-hit mammalian microglia experimental model to mimic multiple perinatal neuroinflammation episodes. The isolation of viable and highly purified microglia populations from *in vivo* LPS-exposed brain allowed an *in vitro* characterization of this cell type. Our most striking discovery was that the fetal inflammatory microglial phenotype acquired during *in vivo* exposure to LPS, even if not histologically apparent, is sustained and potentiated *in vitro* upon re-exposure to LPS. The subsequent RNA sequencing of the microglial genome revealed a unique *HMOX1*_down_ and *FBP*^up^ phenotype of microglia exposed to the double-hit, suggesting interplay of inflammatory and metabolic pathways.

### *In vivo*–*in vitro* model of perinatal inflammation double-hit

Intrauterine exposure to inflammatory stimuli may switch innate immunity cells such as macrophages and microglia to a reactive phenotype (“priming”). Confronted with renewed inflammatory stimuli during labor or postnatally (especially in preterm neonates in the intensive care unit), such sensitized cells can sustain a chronic or exaggerated production of proinflammatory cytokines associated with neurodevelopmental deficits persisting into adulthood (double-hit hypothesis) (Larouche et al., 2005; Spencer et al., 2006; Wang et al., 2006).

Experimentally induced inflammation in chronically instrumented non-anesthetized fetal sheep is a well-established *in vivo* model of fetal physiology (Prout et al., 2010, 2012). Primary microglia cultures in different species have been reported for decades (Stansley et al., 2012). We integrated both *in vivo* and *in vitro* models into a new, hybrid system adding the layer of the whole transcriptome analysis using RNAseq analyses. The chief advantage of the new *in vivo*–*in vitro* model presented here is that it allows us to examine microglia responses to LPS-induced double-hit inflammation *in situ* and *in vitro* on integrative physiological, protein and genomic levels, and in a physiologically and clinically meaningful context. This approach has the potential to uncover hitherto unseen relationships between brain and immune system on different scales of organization in the perinatal stage of development, which might accelerate discovery of new treatment strategies.

*In vivo*, our experimental cohort's morphometric, arterial blood gasses, acid-base status and cardiovascular characteristics were within physiological range and representative for late-gestation fetal sheep as a model of human fetal development near term (Frasch et al., 2007; Rurak and Bessette, 2013). As reported elsewhere (Durosier et al., 2015), the effect of the low LPS dose we administered on the arterial blood gasses, acid-base status and cardiovascular responses is compatible with a mild septicemia (mild compensated metabolic acidemia and hypoxia) evidenced by a transient rise of IL-6 at 3 h without overt shock, i.e., without cardiovascular decompensation. Similar levels of systemic IL-6 have been reported (Prout et al., 2010).

*In vitro*, we developed a new microglia isolation protocol that combines the human adult and fetal brain microglia isolation protocols (Durafourt et al., 2013) and successfully collected a highly enriched microglia population. The use of a modified cell isolation approach from fetal brain tissue is mainly due to the higher degree of myelination in the adult human brain compared to that of a near-term fetus (Durafourt et al., 2013). We were able to attain high purity of microglia, which we validated by flow cytometry and immunocytochemistry. Moreover, RNAseq showed a consistent and constant low level of expression of the astrocyte marker GFAP further confirming cell purity.

In this study, the morphology of microglia from *in vivo* LPS exposed fetal brain was distinguished by more aggregation or clumping and less ramification compared to naïve microglia (Suzuki et al., 2006; Henkel et al., 2009). This suggests that microglia exposed to LPS *in vivo* may have been already activated before plating in cultures.

### Single-hit LPS exposure *in vitro* results in up regulation of inflammatory pathways JAK-STAT and NFKB and down regulation of metabolic pathways

Gene ontology analyses of DE genes in N_L_ microglia revealed an up regulation of inflammatory pathways NFκB, PIK3-Akt, and Jak-STAT. Interestingly, this was accompanied by a down regulation of metabolic pathways in LPS-induced inflammatory response (Figures 3A,B and Table 3). These findings may be explained, at least in part, by the emerging role of energy-sensing AMPK signaling in microglia, which links the inflammatory and metabolic regulatory networks (Frasch, 2014), We will return to this observation in Section Double-hit LPS Exposure of Microglia *In vivo* and *In vitro* Is Uniquely Characterized By a HMOX1_down_/FBP^up^ Phenotype.

Among differentially expressed genes selected, NFKB (log_2_ = 2.676 and $p_{\text{adj}}=4.58\times 10^{-2}$) and JAK3 (log_2_ = 2.965 $p_{\text{adj}}=2.49\times 10^{-3}$) were up regulated in N_L_ microglia. We then investigated the expression of genes involved in the NFκB and JAK-Stat pathways; our data showed that IL1B (log_2_ = 7.578), TNF (log_2_ = 4.990), NFKBIA (log_2_ = 2.578 and $p_{\text{adj}}=9.09\times 10^{-2}$), and RELB (log_2_ = 1.503) were up regulated in N_L_ microglia. Gene ontology analysis revealed down regulation of the energy consuming processes and up regulation of energy conserving processes, as evidenced by the down regulation of genes related to glycolysis (*GPI*) and up regulation of gluconeogenesis (*FBP*) and the insulin signaling pathway. Furthermore, Gene Ontology of up regulated genes revealed that the GO term “immune system process” clustered key genes of inflammatory pathways, such as, JAK3, NFKBIA, and NFKBIB (GO:0002376 and *P* = 9.56 × 10^−8^). Differentially expressed down regulated genes also affected “the immune system process” (GO:0002376 and *P* = 3.24 × 10^−4^) and cellular response to metal ion (GO: 0071248 and *P* = 6.05 × 10^−6^). *HMOX1* and *FOS* clustered in both GO terms underlying the potential role of *HMOX1* in the immune system in relation with *FOS*. Analysis of all down regulated genes showed that the “metabolic process” (GO:0008152 and *P* = 2.38 × 10^−8^) was also globally affected (data not shown).

### Double-hit LPS exposure of microglia *in vivo* and *in vitro* is uniquely characterized by a HMOX1_down_/FBP^up^ phenotype

Interestingly, *HMOX1* gene expression showed a strong down regulation in SH_L_ and SH_C_ by four-fold (Table 3, Figures 3D,E). The level of expression of *HMOX1* was higher in SH_C_ than in SH_L_ (log_2_ = −4.462 and $p_{\text{adj}}=4.22\times 10^{-19}$; log_2_ = −4.303 and $p_{\text{adj}}=8.13\times 10^{-2}$, respectively). Such differences in response patterns were observed in other genes as well suggesting a memory of inflammation induced by pre-exposure to LPS *in vivo*.

*HMOX1* role in microglia is yet to be fully understood. Across the three group comparisons (Figure 1A), *HMOX1* was significantly down regulated and *in vivo* pre-exposure to LPS seemed to further enhance the down regulation of *HMOX1* in response to second-hit *in vitro* LPS stimulation. We confirmed by RT-PCR that transcript amounts of *HMOX1* are low in N_L_ and SH_L_ microglia (Figures 4A,B). *HMOX1* was suggested to play an anti-inflammatory role in LPS-induced murine adult cell line macrophages via the activation of the Nrf2/ARE pathway (Ye et al., 2014). Pre-treatment with Oroxylin A, an inhibitor of LPS-induced mRNA, substantially increased the levels of *NRF-2* and heme oxygenase 1. The response of SH_C_ and SH_L_ compared to their single-hit microglia counterpart N_L_ showed that *HMOX1* and *NRF-2* had a greater down regulation after pre-exposure to LPS *in vivo* (Table 3), supporting the potential role of *HMOX1* in the inflammatory response and as a determinant of microglial phenotype.

While the role of *FBP* in inflammation is unclear, its neuroprotective effect in brain injury models was suggested through various mechanisms. During hypoxia, *FBP* supports ATP production via stimulation of glycolysis which results in maintenance of normal intracellular calcium levels via the phospholipase-C dependent MAP kinase signaling pathway (Bickler and Kelleher, 1992; Fahlman et al., 2002). When comparing gene expression in SH_C_ (Figures 3C,D), we observed that genes responsible for the transport of calcium, potassium and second messengers were also differentially expressed and up regulated.

It was previously reported that FBP dose-dependently suppressed LPS-induced nitric oxide (NO) production, and higher FBP doses were also associated with decreased levels of the transcription factor activator protein AP-1 in primary neonatal murine microglia cultures (Kim et al., 2012). We confirmed this observation in our comparison of SH_L_ to SH_C_, wherein we observed that up regulation of *FBP* was concordant with lower expression of *NOS1AP* (log_2_ = −1.393) suggesting lower production of NO. We also observed that lower FBP transcripts amount in N_L_ was accompanied by higher expression level of NOS production related genes (Table 3). The authors observed that FBP had an effect on the binding of transcription factors to DNA: FBP diminished the binding of AP-1 to DNA, but NFKB and CREB did not seem affected. We found down regulation of *AP-1* (log_2_ = −2.385) and a slight down regulation of *NFKB* and *CEBP*, though *CREB1* remained unaffected. In the SH_L_, our results confirmed that DNA binding nuclear factors were not strongly down regulated upon higher transcript level of *FBP*. The authors suggested that FBP inhibits iNOS expression by blocking the JNK/p38 MAPK pathway. We confirmed that *JNK* related genes may have lower expression level, however we did not observe any marked difference for *P38*.

A common theme within the newly found *HMOX1*_down_*/FBP*^up^ phenotype appears to be its memory of the “energy restoring direction” following *in vivo* exposure to LPS. This metabolic effect is evidenced for example by up regulation of AMPK, insulin, growth arrest processes, mitochondrial biogenesis signaling pathways and down regulation of mTOR signaling pathway and such energy consuming processes as cell growth and protein synthesis (cf. Table 3).

### Does *in vivo* endotoxin exposure induce transcriptome memory of inflammation in fetal microglia mediated by epigenetic mechanisms?

Pre-exposure to LPS *in vivo* affected globally the transcriptome of microglia (Table 3). We observed that SH_C_ microglia had a diminished response in gene expression of inflammatory pathways NF-κB, JAK-Stat, and PIK3-Akt compared to the behavior of the N_L_ microglia; this phenomenon was sustained in SH_L_ microglia. As mentioned in Section Double-hit LPS Exposure of Microglia *in vivo* and *In vitro* Is Uniquely Characterized By a HMOX1_down_/FBP^up^ Phenotype, this was also true for the metabolic pathways. This desensitization was also observed in histone deacetylase 1 (*HDAC1 and 6*), which was DE up regulated by two-fold in N_L_ (log_2_ = 2.271 and $p_{\text{adj}}=8.58\times 10^{-2}$), and up regulated by less than one-fold in SH_C_ and SH_L_ (log_2_ = 0.676 and log_2_ = 0.145, respectively, Table 3). This *HDAC1* profile was accompanied by a less than one-fold down regulation of *HDAC6* (log_2_ = −0.688 and $p_{\text{adj}}=8.75\times 10^{-2}$) followed again by desensitization in microglia exposed to LPS *in vivo*. *HDAC4* was 2.5-fold up regulated (log_2_ = 1.292 and *p*_adj_ = 0.133) in N_L_ vs. N_C_ microglia followed also by desensitization in the comparison to the *in vivo* pre-exposed microglia; meanwhile, *HDAC2* showed a less than one-fold down regulation (log_2_ = −2.746 and $p_{\text{adj}}=2.30\times 10^{-4}$) in N_C_ vs. SH_C_ microglia, with no detectable change in microglia exposed to LPS *in vitro* only or upon double-hit exposure. In parallel, *HMOX1* was down regulated by four-fold in SH_L_ and SH_C_, and by two-fold in N_L_ microglia, and did not seem to have a diminished response in SH_L_. These findings underscore the potential role of *HDAC1, 2, 4*, and *6* in the memory of the *in vivo* exposure to inflammation in line with the histone code hypothesis (Jenuwein and Allis, 2001).

In light of the putative epigenetic mechanisms underlying our findings of single- and double-hit LPS signatures in microglial transcriptomes, it remains to be tested whether these signatures are indeed unique to LPS or apply more widely for perinatal exposures to other stressors, such as the psychosocial stress, e.g., caused by fear (Shapiro et al., 2013; Monteleone et al., 2014; Metz et al., 2015). Forced-swim stress applied over 4 days in adult male mice induced changes lasting at least two following weeks in neuronal acetylcholine esterase (AChE) expression via an epigenetic mechanism of hypoacetylation, with near-exclusive enrichment of HDAC4, and hypermethylation of histone H3K9 at a specific promoter of AChE (mP1c) with resulting suppression of the mE1c exon expression levels (Meshorer et al., 2002; Sailaja et al., 2012). Interestingly, a non-exclusive increase of HDAC-1, 2, and 7 was also detected. Animals showed anxiety-like behavior and this behavior as well as the AChE chromatine structure and the entire HDAC enrichment profile were reversed by NaBu, an HDAC inhibitor; the restoration of mE1c expression level was however due to HDAC4 inhibition entirely. AChE-R is the alternative splicing soluble variant of AChE-S in neurons; AChE-R production increases under various stress influences (Soreq and Seidman, 2001). This splicing switch can be induced by short-lasting (minutes) stress exposures, but can then last for weeks as shown in adult neuronal and hippocampal slice cultures (Meshorer et al., 2002; Sailaja et al., 2012). NaBu restored this splicing switch with regard to reduction in AChE-R, although the renewed increase of AChE-S variant was incomplete compared to the non-stressed animals. This finding is particularly interesting, as it sheds a new light on how stress may modulate inflammation via epigenetic mechanisms impacting the pro-inflammatory AChE. AChE inhibition restricts inflammation not only in the peripheral organs, but also in the brain (Pollak et al., 2005). The incomplete restoration of AChE-S suggests a complex regulatory network controlling AChE-S/AChE-R ratio in response to stress. Ultimately, such shifts in AChE presence in intercellular space may have long-lasting effects on cholinergic transmission with regard to cognition (cf. Section Microglial LRP-mediated Neuronal Phagocytosis May Be Enhanced By *In utero* Exposure to Inflammation) and neuroinflammation. Adding to the complexity of epigenetic regulation of cholinergic signaling and neuroinflammation, microRNA (miRNA)-132 has been shown in adult murine model and cell lines to potentiate cholinergic anti-inflammatory signaling in the periphery, myeloid cells in particular, and in the brain by inhibiting AChE expression (Shaked et al., 2009). The role of miRNA-132 in microglia is not yet known, but the evidence is growing for the overall importance of miRNA signaling in determining the polarization and phenotype of microglia and myeloid cells in general (Ponomarev et al., 2013).

Fear represents a model system to study chronic impact of stress on epigenome and cardiovascular system (Shenhar-Tsarfaty et al., 2015). As noted above, this approach may relate conceptually to our current findings bringing together the effects of *in vivo* endotoxin exposure as a stressor on the brain's microglial transcriptome and the cardiovascular system. Interestingly, changes in miRNA-608 activity on AChE binding sites in the brain (e.g., due to single nucleotide polymorphisms, SNPs) concomittantly raise levels of anxiety and blood pressure in adult mice and humans by decreasing the inhibition of AChE expression, while reducing CDC42 and IL-6 levels, important pro-inflammatory mediators (Hanin et al., 2014). This link between epigenetic signaling mechanisms, stress, and cardiovascular system is further strengthened by the recent study, in adult humans showing synergistic effects of fear as a stressor on heart rate and inflammation with cholinergic signaling playing a central role in modulating both systems (Shenhar-Tsarfaty et al., 2014, 2015). We found an increase of heart rate and a slight drop of blood pressure within the time frame of the IL-6 peak following LPS injection to the ovine fetus (Durosier et al., 2015). However, this effect appeared to dissipate at 54 h following the initial LPS exposure. Still, our experimental design does not allow drawing conclusions whether such intrauterine exposure to low-dose endotoxin concentrations may induce lasting cardiovascular changes along with alterations in innate immune responses upon repeated exposure to inflammatory stimuli. This remains subject of future studies. Interestingly, *BCHE*, but not *ACHE*, showed DE and less than one-fold down regulation (log_2_ = −3.197 and $p_{\text{adj}}=6.86\times 10^{-3}$) in N_C_ vs. SH_C_ microglia, with no detectable change in microglia exposed to LPS *in vitro* only or upon double-hit exposure; both *BCHE* and *ACHE* were also less than one-fold down regulated, but not differentially expressed in all other comparisons. In this regard, the potential role of serum cholinesterases as easily accessible biomarkers of neuroimmune function, along with heart rate variability monitoring, present an attractive opportunity to translate these insights into bedside applications to improve perinatal health outcomes (Durosier et al., 2015; Lake et al., 2014; Shenhar-Tsarfaty et al., 2014).

In summary, microglia pre-exposed to inflammation *in vivo* seem to acquire a memory of inflammation that reflects on the transcriptome by an overall decreased response in inflammatory pathways while the production of the pro-inflammatory cytokine IL-1β is up regulated. In light of the above discussion, our findings lend support to the notion of an inflammation memory in SH_C_ sustained in SH_L_ microglia that may be mediated by epigenetic regulatory processes involving histone acetylation and miRNA signaling. The intriguing link to the metabolic processes and cardiovascular system also deserves attention in future studies. Additional mechanistic studies (knockout, knockdown, or overexpression) are needed to validate these observations.

### Microglial LRP-mediated neuronal phagocytosis may be enhanced by in utero exposure to inflammation

Calreticulin (CRT) exposure on the surface of viable or apoptotic neurons is required for their phagocytosis via low-density lipoprotein receptor-related protein (LRP) receptors on LPS-stimulated primary culture rat microglia (Fricker et al., 2012). We found that the gene *LRP6* is significantly up regulated after LPS exposure *in vitro* in N_L_ microglia (log_2_ = 1.052 and $p_{\text{adj}}=2.76\times 10^{-2}$) and the activation of *LRP6* is sustained *in vitro* in SH_C_ microglia (log_2_ = 1.850 and $p_{\text{adj}}=5.58\times 10^{-3}$), i.e., after the LPS exposure *in vivo*. *LRP1B* (log_2_ = 6.380 and $p_{\text{adj}}=5.66\times 10^{-5}$) and *LRP2* (log_2_ = 6.571 and $p_{\text{adj}}=4.24\times 10^{-11}$) were also strongly up regulated in SH_C_ microglia. *LRP1B* and *LRP2* showed a four-fold up regulation in N_L_ microglia, however, adjacent *p*-values were not consistent to support this observation.

We show that a single LPS exposure *in vivo* or *in vitro* suffices to up regulate *LRP* genes suggesting that *in utero* exposure to inflammation may alter microglial—neuronal communication making CRT expressing neurons vulnerable to LRP-mediated phagocytosis. Our data does not allow validating the idea that double hit exposure to an inflammatory stimulus enhances up regulation of microglial *LRP*, because we could not test directly SH_L_ vs. SH_C_ (cf. Section Methodological Considerations, Discussion on limitations of RNAseq approach). Future studies, should estimate genetic expression profile of SH_L_ compared to SH_C_.

### Methodological considerations

We could not detect any *in situ* neuroinflammation using Iba1, a well-established myeloid cell marker in sheep and other species. Despite the lack of overt neuroinflammation seen *in situ* we demonstrated a pattern of LPS-induced systemic IL-6 cytokine production *in vivo* and microglial IL-1β cytokine secretion *in vitro*. This further supports the notion that even subtle LPS exposures *in utero in vivo* may polarize microglia toward a neuroinflammatory phenotype without or with secondary re-exposure to an inflammatory stimulus. The LPS-triggered rise of IL-6 in plasma is in line with animal and human studies at this developmental stage (Duncombe et al., 2010; Chan et al., 2013). Microglia *in vitro* have been shown to secrete IL-1β preferentially when challenged with LPS, while IL-6 secretion is a hallmark of cultured astrocytes in rat (Gottschall et al., 1994). Our findings are consistent with literature and further support the cell culture purity.

In parallel to our team, the feasibility of creating a mixed primary fetal ovine brain culture has been recently, demonstrated (Weaver-Mikaere et al., 2012). We have advanced this work by focusing on late rather than mid-gestation fetuses and creating primary pure microglial culture rather than mixed culture. This allowed us to then study the microglia-specific effects of the double hit *in vivo/in vitro* LPS exposure on the secretion profile of the inflammatory cytokine IL-1β and the high-throughput transcriptome.

In this study, we did not discriminate between the various phenotypes of the endogenous microglia as well as the microglia recruited to the brain during the inflammatory process via the blood brain barrier, whose permeability increases under conditions of hypoxia/ischemia and fetal inflammatory response (Hutton et al., 2007, 2008; Butovsky et al., 2014; Yamasaki et al., 2014; Greter et al., 2015; Sadowska et al., 2015). Considering the mild, low-dose LPS exposure, we speculate that no recruitment of peripheral monocytes was triggered. However, we cannot state with certainty whether the microglial memory of inflammation was entirely newly established upon *in vivo* LPS exposure, or certain pre-existing sub-populations of microglia responded differentially to the endotoxin; another possibility needing validation remains that progenitor cells from the periphery differentiated accordingly. Hence, future studies, perhaps using single cell RNAseq, will elucidate whether the “memory” is entirely newly established, carried by a subpopulation of endogenous or periphery-recruited microglia. Isolating single cells and expanding them in culture may be another approach to test these hypotheses.

In our approach, we used DESeq2 to normalize read counts and identify differentially expressed genes. DESeq2 was specifically designed to estimate differential expression in a dataset containing replicates for both control and treatment samples. Our method used a large number of animals allowing us to have replicates for naïve control and LPS-exposed microglia. However, a limitation of our RNAseq analysis is the lack of replicates for SH_C_ and SH_L_ preventing us from comparing SH_C_ to SH_L_ directly. Other platforms meant to analyze samples without replicate could have been used here. However, we chose not to disrupt the analytical pipeline and keep the statistical analysis consistent throughout the analysis. Despite quality control measures prior to sequencing, the sample N_L3_ had a different expression pattern than the two other N_L_ samples. We believe it is not related to RNA quality, and may have been due to environmental or other physiological conditions of the animal that we were not aware of at the time of the experiment. In interrogating the differential gene expression, we have ensured that the partially deviating pattern observed in sample N_L3_ did not confound our findings (Figures 3A,B).

## Conclusions

Inflammatory microglial phenotype acquired during *in vivo* exposure to LPS is sustained and potentiated *in vitro* upon re-exposure to LPS. We identified a unique *HMOX1*_down_ and *FBP*^up^ phenotype of microglia exposed to the double-hit. Our results also suggest that microglia may have acquired *in vivo* a memory of inflammation regulated by an epigenetic process that should be confirmed by further epigenomic studies. This model allows studying mechanisms of fetal neuroinflammation *in utero in vivo* and *in vitro* to identify potential therapeutic targets for early postnatal intervention to prevent brain injury.

### Conflict of interest statement

The authors declare that the research was conducted in the absence of any commercial or financial relationships that could be construed as a potential conflict of interest.
