# Supplementary material for: Fetal microglial phenotype in vitro carries memory of prior in vivo exposure to inflammation
Source: Front Cell Neurosci. 2015 Aug 4;9:294. doi: 10.3389/fncel.2015.00294 (PMC4524165; doi:10.3389/fncel.2015.00294)
Supplement: Supplementary file 1 [file DataSheet1.DOC]

**Fetal microglial phenotype *in vitro* carries memory of prior *in vivo* exposure to inflammation**

**M. Cao^1*^, M. Cortes^2*^, C.S. Moore^3^, S.Y. Leong^3^, L.D. Durosier^1^, P. Burns^4^, G. Fecteau^4^, A. Desrochers^4^, R.N. Auer^5^, L. B. Barreiro^6^, J.P. Antel^3^, M.G. Frasch^1,2^**

* M. Cao and M. Cortes contributed equally to this manuscript

*^1^Dept. of Obstetrics and Gynaecology and Dept. of Neurosciences, CHU Ste-Justine Research Centre, Faculty of Medicine, ^2^Animal Reproduction Research Centre (CRRA), Faculty of Veterinary Medicine, Université de Montréal, Montréal, QC, Canada;*

*^3^Neuroimmunology Unit, Montréal Neurological Institute, McGill University, Montréal, QC, Canada;*

*^4^Dept. of Clinical Sciences, Faculty of Veterinary Medicine, Université de Montréal, QC, Canada*

*^5^University Hospital Ste-Justine, Département de Pathologie, Université de Montréal, QC, Canada*

*^6^Dept. of Pediatrics, CHU Ste-Justine Research Centre, Faculty of Medicine, Université de Montréal, Montréal, QC, Canada*

**Address of correspondence:**

Martin G. Frasch

CHU Ste-Justine Research Centre

Dépt. obstétrique-gynécologie, Université de Montréal

3175, Côte-Sainte-Catherine

Montréal (Québec)

H3T 1C5, Canada

Phone: +1-514-345-4931 x4048

Fax: +1-514-345-4801

Email: [martin.frasch@recherche-ste-justine.qc.ca](mailto:martin.frasch@recherche-ste-justine.qc.ca)

**Abbreviated title:** Fetal microglia *in vivo* and *in vitro*

**Supplemental Materials**

1. Cell purity
   1. *Flow Cytometry*

After several days in culture, sheep microglia were scraped from the wells using a cell scraper and blocked for 30 minutes using normal mouse IgG (3 µg/ml) and 10% human serum. Cells were then stained using a FITC-conjugated monoclonal bovine anti-CD11b (1:40, Bio-Rad) on ice for 20 minutes. Cells were washed in FACS buffer at 1200rpm for 5 min and fixed with 1% PFA and acquired using a FACSCalibur flow cytometer (BD Biosciences), data was analyzed using FlowJo software (Tree Star, San Carlos, CA).

- 1. *Immunocytochemistry (ICC) analysis*

After media removal, cells cultured in chamber slides were fixed with 1% formaldehyde for 10min, followed by gently washing 3 times 5min each with PBS, then blocked with PBST (PBS with 0.3% Triton-100) for 20min. Cells were incubated for 1h in the dark with rabbit anti-human Iba1 (a microglia marker, Wako Cat no. 019-19741) antibody at a dilution of 1: 250, together with Hoechst (Invitrogen H3569, dilution 1:5000), washed 3 times for 5min. Cells were incubated with Alexa 568 (goat anti-rabbit, Life Tech Cat no A11036, dilution 1: 400) for 1h, after wash, mounting with Fluoromount-G (SouthernBiotech, Cat no 0100-01) before imaging. Control wells of astrocytes were incubated with GFAP-Cy3 (Sigma C9205) at a dilution of 1:500. Images were captured by an immunofluorescence inverted microscope (Carl Zeiss Axiovert 200).
